# The biological roles of CD47 in ovarian cancer progression

**DOI:** 10.1007/s00262-024-03708-3

**Published:** 2024-06-04

**Authors:** Linan Xing, Zhao Wang, Yue Feng, Haixia Luo, Guijiang Dai, Lin Sang, Chunlong Zhang, Jianhua Qian

**Affiliations:** 1 Department of Gynecology, The First Affiliated Hospital, Zhejiang University School of Medicine, Hangzhou, 310003 People’s Republic of China; 2 College of Bioinformatics Science and Technology, Harbin Medical University, Harbin, 150081 People’s Republic of China; 3 Department of Gynecological Oncology, Zhejiang Cancer Hospital, Hangzhou Institute of Medicine (HIM), Chinese Academy of Sciences, Hangzhou, 310022 People’s Republic of China; 4 Department of Comprehensive Office, The Second Affiliated Hospital of MuDanjiang Medical University, Mudanjiang, 157009 People’s Republic of China; 5 Department of Obstetrics and Gynecology, People’s Hospital of Anji, Huzhou, 310022 People’s Republic of China

**Keywords:** CD47, Ovarian cancer, Sirpα, TSP-1, Immunotherapy

## Abstract

Ovarian cancer is one of the most lethal malignant tumors, characterized by high incidence and poor prognosis. Patients relapse occurred in 65–80% after initial treatment. To date, no effective treatment has been established for these patients. Recently, CD47 has been considered as a promising immunotherapy target. In this paper, we reviewed the biological roles of CD47 in ovarian cancer and summarized the related mechanisms. For most types of cancers, the CD47/Sirpα immune checkpoint has attracted the most attention in immunotherapy. Notably, CD47 monoclonal antibodies and related molecules are promising in the immunotherapy of ovarian cancer, and further research is needed. In the future, new immunotherapy regimens targeting CD47 can be applied to the clinical treatment of ovarian cancer patients.

**Supplementary Information:**

The online version contains supplementary material available at 10.1007/s00262-024-03708-3.

## Introduction

Ovarian cancer is one of the three gynecologic cancers with the highest mortality rate. The symptoms and signs of ovarian cancer disease are atypical, and there is a lack of specific screening tools. Most ovarian cancer patients are diagnosed at an advanced stage, and their 5-year overall survival (OS) rate is only 47% [1]. It has the characteristics of high recurrence rate, low diagnostic rate and poor prognosis, which seriously threatens women’s life and health [2]. Nowadays, ovarian cancer cytoreductive surgery, platinum-based chemotherapy, finally maintenance therapy with bevacizumab and Poly ADP-ribose polymerase (PARP) inhibitors are the main treatments for ovarian cancer [3, 4]. However, about 65–80% of advanced ovarian cancer patients will experience recurrence [5]. Eventually, with the increase of chemotherapy cycles, platinum-sensitive recurrent cancers will become resistant to platinum. This is also the main reason for the poor prognosis of patients with advanced ovarian cancer [6]. Therefore, improving the survival time and prognosis of ovarian cancer patients is an urgent clinical need in the gynecological oncology community.

**Fig. 1:**
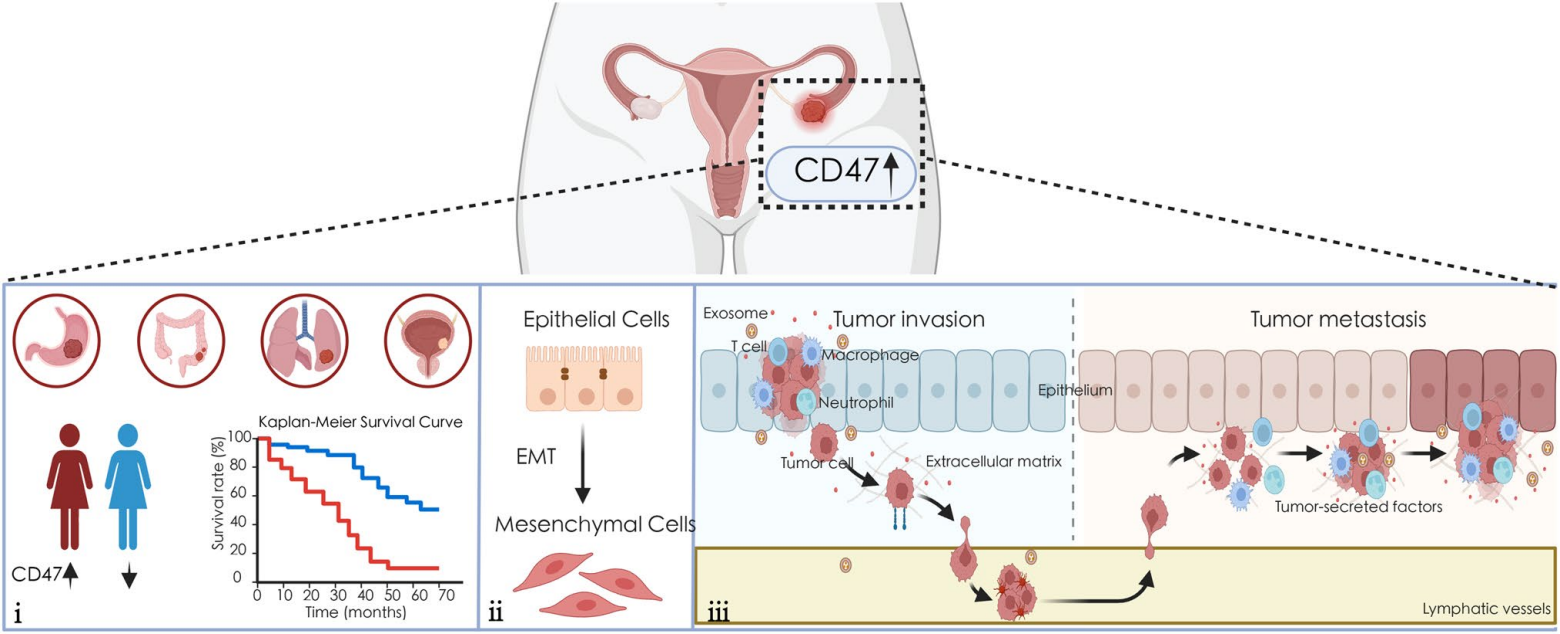
CD47 is considered as a diagnostic marker for ovarian cancer. **i** CD47 is overexpressed in ovarian cancer and a variety of tumor tissues. Patients with high CD47 expression have worse survival. **ii** Overexpression of CD47 can induce EMT. **iii** Overexpression of CD47 can promote the invasion and migration of ovarian cancer cells

CD47 is a cell surface glycoprotein of the immunoglobulin superfamily, formerly known as integrin-associated protein (IAP) [7]. It consists of an extracellular N-terminal IgV domain, five transmem-brane domains and a short C-terminal cytoplasmic tail. These three domains are variable between humans and animals, so it has four alternative isoforms [8]. It can bind to a variety of proteins, including integrin, thrombospondin-1 (TSP-1), and signal regulatory protein alpha (Sirpα) (Fig. 1 in Supplementary material) [9]. As shown in Fig. 1, we learn that CD47 is ubiquitously expressed in normal and malignant tissues [10]. And its expression is increased in a variety of tumor tissues, including ovarian [11], gastric cancer [9], colorectal cancer [12], lung squamous cell carcinoma [13], bladder tumor [14], etc. It can participate in a variety of cellular functions and plays an important role in them, including proliferation, apoptosis, adhesion, migration and a variety of immune responses [8, 15–17]. In general, high CD47 expression is associated with poor prognosis. Taken together, it is usually employed as a marker to judge the prognosis of cancer patients.

## CD47: a marker for ovarian cancer diagnosis

**Fig. 2:**
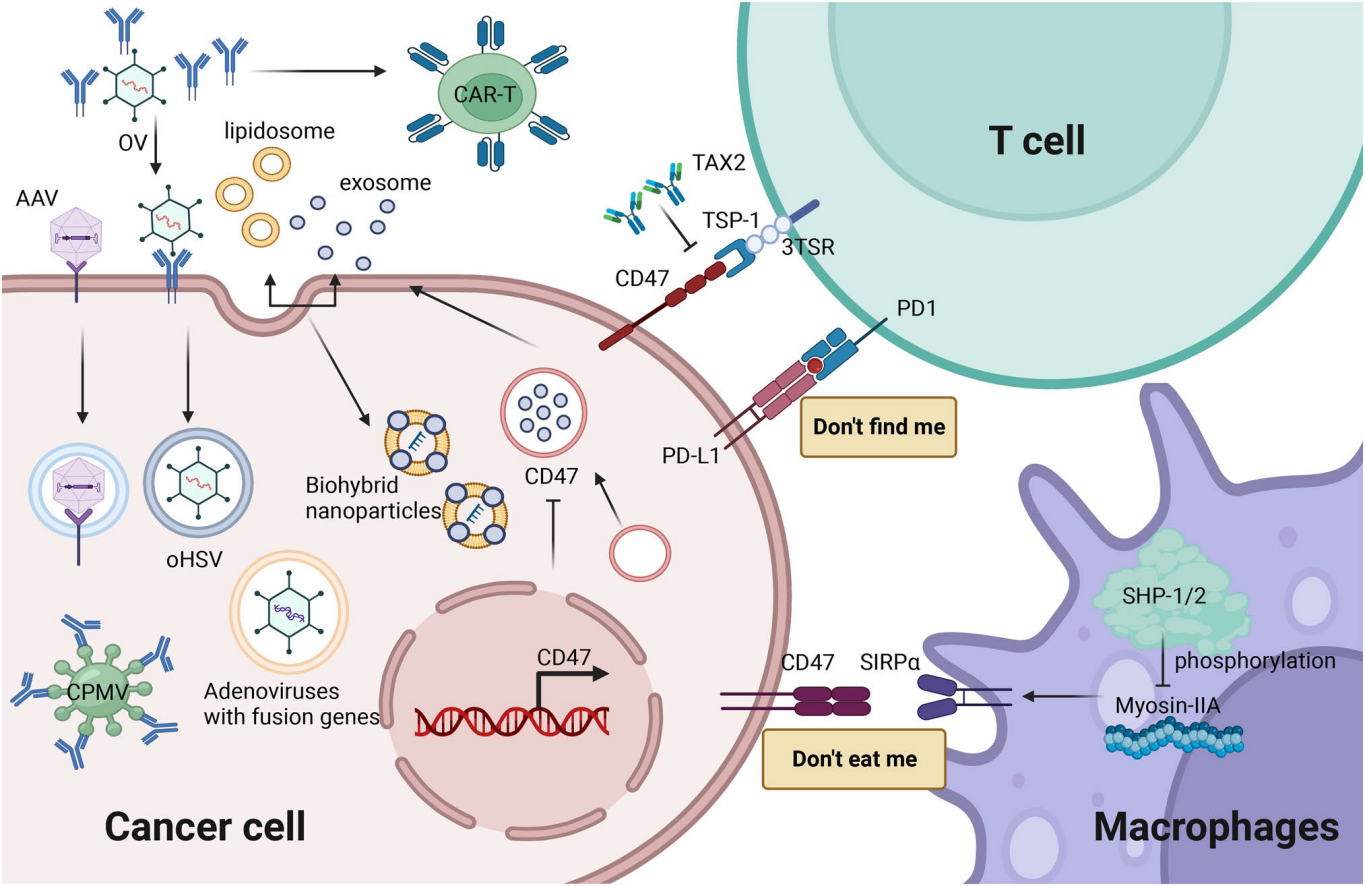
The mechanism of action of CD47 in ovarian cancer. The interaction between CD47 and Sirpα recruits SHP-1/2, leading to phosphorylation of both. In turn, they block myosin IIA accumulation, inhibit phagocytic synapse formation, and finally send a “don’t eat me” signal. Inhibition of exosome secretion and uptake can inhibit the expression of CD47 on the surface of ovarian cancer cells and enhance the phagocytosis of macrophages. The CD47/Sirpα pathway could be blocked by the combination of antibodies secreted by oHSV infected tumor cells and anti-CD47 mAb, as well as by the construction of fusion gene oncolytic adenovirus. Biohybrid nanoparticles formed by the fusion of engineered exosomes transfected with CD47 gene and liposome membrane can deliver drugs and avoid clearance by MPS system, induce apoptosis of ovarian cancer cells and overcome drug resistance. TSP-1 contains 3TSR. CD47 is the receptor for TSP1 signaling expressed on T cells. AAV-mediated expression of 3TSR can inhibit tumor progression. The combination of CD47 Ab and CPMV has a synergistic potential to induce tumor cell death through activation of macrophages. CD47-CAR-T cells only affect the highly expressed CD47 cells and have highly effective anticancer properties. The in situ antagonist of TSP-1, TAX2, has anticancer properties and is able to be selective for TSP-1/CD47 interaction. Created with Biorender

CD47 is commonly overexpressed in ovarian cancer patients and is associated with poor prognosis [18–20]. It was elevated in 90% of ovarian cancers and rarely detected in normal tissues. Therefore, it has been identified as an ovarian tumor marker, known as OA3 (Fig. 2 in Supplementary material) [21, 22]. However, it has been recognized that CD47/OA3 is more widely distributed in normal adult tissues than previously appreciated. The differential gene expression in TCGA database also confirmed that the CD47 expression encoding gene was up regulated in ovarian cancer patients. Moreover, upregulation of CD47 expression was associated with worse OS and progression free survival (PFS) in ovarian cancer [23]. It has been reported that CD47 is amplified in 15/316 (5%) of TCGA ovarian serous carcinomas. In the validation cohort, the majority of patients had stage III/IV disease (208 of 265,78.4%) and was expressed in 210 of 265 (79.2%). In that study, patients with tumors with low versus high CD47 expression had a higher rate of complete response to adjuvant therapy (65% vs. 50%, *p* = 0.026). Although there was a trend toward increased median OS in the CD47 low expression group compared with the high group (37.64 vs. 45.26 months, *p* = 0.92), the difference was not significant (Fig. 2) [24]. In previous studies, immunohistochemical analysis presented that CD47 expression level in ovarian cancer tissues and borderline tumors was significantly higher than that in benign tumors and normal tissues. The high CD47 expression was significantly correlated with the grade, lymph node metastasis and differentiation in ovarian cancer. Cox model and survival analysis manifested that high CD47 expression was an independent poor prognostic factor for ovarian cancer (Fig. 2 in Supplementary material) [25]. In ovarian cancer cell model, overexpression of CD47 significantly promoted migration and invasion. In addition, CD47 can also induce epithelial-mesenchymal transition (EMT) by regulating E-cadherin and N-cadherin (Fig. 2 in Supplementary material) [26].

Ovarian endometriosis can lead to malignant epithelial ovarian tumors [27], which is a special pathological type of epithelial ovarian cancer [28]. Wang and Lin et al. collected 36 clinical ovarian samples, used immunohistochemistry to detect the protein expression profile of CD47 and analyze its correlation with clinicopathological features. It was found that the CD47 expression was relatively high in patients with endometriosis-associated ovarian cancer (EAOC) compared with the normal group. The high expression of CD47 was positively correlated with histological type (*p* = 0.007) and tumor grade (*p* = 0.002). In the TOV-112D and TOV-21G cell lines, CD47 overexpression promoted cancer cell growth and motility. Similarly, silencing CD47 and anti-CD47 monoclonal antibody (anti-CD47 mAb) exerted an inhibitory effect on tumor cells (Fig. 2 in Supplementary material) [29]. The occurrence of clear cell carcinoma (OCCC) is closely related to endometriosis. Some researchers employed immunohistochemical methods to detect the expression of CD44, CD47 and c-met in 86 OCCC cases. Patients with low expression levels had a higher survival rate than those with high levels. The high expression of CD44/CD47 and CD47/c-met was also correlated with the above factors (surgical stage, chemotherapy resistance and prognosis), but not with lymph node metastasis. This study’s results pointed out that CD47 expression was an independent risk factor for the prognosis of OCCC (Fig. 2 in Supplementary material) [11].

Recurrence of ovarian cancer and resistance to current chemotherapy regimens are a global challenge. Cancer stem cells (CSCs) are a subpopulation of tumor cells that are associated with drug resistance and tumor recurrence. Its proportion is small, and it always maintains tumor growth and heterogeneity in the process of tumor recurrence. Thus, eliminating ovarian CSC can be regarded as an effective treatment to reduce chemotherapy resistance and recurrence of ovarian cancer [30]. Acetaldehyde dehydrogenase (ALDH) is another important mechanism of CSC drug resistance [31, 32]. ALDH overexpression is considered a prognostic marker in many cancers [33–36], including ovarian cancer [37]. Recent reports proved that high ALDH1 expression (ALDHigh) can be used as a marker for CSC on the ovary [38, 39]. Sharrow and Perkins et al. analyzed the CSC properties of ALDHigh ovarian cancer cells. The FNAR-C1 and SKOV3 ovarian cancer cell lines were tested. It was found that ALDHigh cells in both models exhibited phenotypic, biological and functional stem cell properties. Moreover, CD47 expression was approximately 2-fold higher in ALDHigh cells than in low expression cells in both models, as measured by microarray and qPCR. Therefore, the authors suggested that CD47 may be a target for ovarian CSC in the future (Fig. 2 in Supplementary material) [40]. A variety of FGFR3 inhibitors and antibodies targeting CD47 are now being tested in clinical trials. Accordingly, it is necessary to determine the best targeted therapy regimen and validate it in primary human tumors for future clinical work. A different picture has been shown in studies in which CSCs are more susceptible to immune surveillance. In this study, researchers isolated mouse and human ovarian cancer stem-like cells from mouse and human cell lines, respectively. C47 protein was found to be low expressed in two ovarian cancer stem-like cells. Stem cell antigen (SCA)-1^+^ ID8 and CD133^+^ HM-1 cells isolated from mouse cancer stem cell-like cells were more susceptible to phagocytosis by macrophages and produced CD8^+^ T cell immunity. SCA-1^+^ ID8 cells were able to grow in syngeneic mice but were quickly rejected. Cancer stem-like cells can only grow in mice when mixed with non-stem cells and were protected from immune attack. This study indicated that ovarian cancer stem-like cells are susceptible to phagocytosis by macrophages due to low CD47 expression. However, the surrounding differentiated bulk tumor cells can protect it from immune clearance and maintain its existence. This revealed that differences in CD47 expression levels may be due to multiple factors in stem-like cells of nest-cancer cells. CD47 expression has a dynamic change in cells with different stem cell states (Fig. 2 in Supplementary material) [41].

## The associations of CD47 with the tumor microenvironment of ovarian cancer

Tumor microenvironment (TME) is a complex structure composed of heterogeneous tumor cell population, a variety of resident and infiltrating host cells, secretory factors, extracellular matrix proteins, extracellular vesicles, and vaso-lymphatic network [42, 43]. The characteristics of malignant cells and the behavior of the whole TME can determine the tumor progression [44–46]. Ovarian cancer is a highly heterogeneous tumor, and the TME is diverse [47, 48]. Immune cell populations are important participants in tumor therapy [49]. In order to better understand ovarian cancer progression, metastasis, and drug resistance, it is necessary to find out the ovarian TME’s function as well as immune cells’ activity and properties. To explore CD47’s effect on ovarian cancer TME, Yu and Ding et al. employed two different data sets from the Tumor Immune Single-cell Hub (TISCH) single cell database to detect the immune cells' distribution at the site of primary quantitative metastatic ovarian tumors. They have presented evidence implicating that the CD47 expression in immune cells was different in tumor ascites of patients with primary and metastatic ovarian cancer. Its expression in tumor ascites of patients with primary ovarian cancer was relatively low in plasma cells, dendritic cells, and single/giant cells. Moreover, the type of immune cells and the degree of CD47 expression in immune cells were different between the two data set. Therefore, they considered that TME in primary and metastatic ovarian cancer may be different, which probably the cause of the ovarian cancer’s heterogeneity (Fig. 2 in Supplementary material) [18].

Tumor infiltrating immune cells (TIIC) are an important tissue part of TME [50]. In the process of tumorigenesis, TIIC can affect immunosuppression and immune evasion to regulate tumor growth. Therefore, quantitative analysis of TIIC different types is helpful to elucidate the mechanism of immune response during tumorigenesis, development and treatment. At the same time, it can provide effective strategies for tumor immunotherapy [51]. The researchers speculate that TIIC may be related to the prognosis of cancer patients [52, 53]. However, there are limited studies on the role of CD47 in immune invasion of ovarian cancer. For example, the research pointed out that the expression level of CD47 was closely related to the immune infiltration of ovarian cancer, especially the positive correlation with the failure of Treg cells, monocytes, macrophages and T cells. The TIMER database was used to analyze the infiltration distribution of different tumor immune cells in ovarian cancer. The results revealed that the invasion of M2 and Tregs was higher in the ovarian cancer microenvironment with high CD47 expression level. Combined with previous studies, it was found that M2 and Tregs can generate immune barrier and instantaneous immune response. Simultaneously, T cell failure will lead to immune escape. Therefore, it can be speculated that high CD47 expression will promote immune escape of ovarian cancer (Fig. 2 in Supplementary material) [18].

Exosomes are cell-derived membrane vesicles (30–200 nm in diameter) with good biocompatibility, little immunogenicity, long circulation, and nontoxicity [54, 55]. It plays a key role in mediating communication between cells and regulating immune response [56, 57]. A growing number of studies have described the relationship between exosomes and tumor development, and validated the role of exosomes in regulating cancer therapy resistance, metastasis, and immunity. In recent years, it has been considered as the key signal medium for regulating TME. Exosomes can reflect their cell origin and disease status through the bioactive substances they transport. Thus, it can become a potential biomarker for disease diagnosis and prognosis, as well as a potential target for cancer treatment [58, 59]. Shimizu and Sawada et al. collected exosomes from ovarian cancer cell lines to detect the expression of CD47 on the exosome surface. Figure 2 shows that inhibition of exosome secretion and uptake can inhibit the CD47 expression on the surface of ovarian cancer cells, and it was found to promote phagocytosis of macrophages. In a xenograft mouse model, they observed if the release of tumor-derived exosomes was knocked down or inhibited, tumor progression would be inhibited. In addition, the phagocytosis of M1 macrophages was enhanced in ovarian tissue. Finally, they have demonstrated that CD47 is expressed on exosomes, and inhibition of exosome secretion and/or uptake can enhance the phagocytosis of macrophages, thereby inhibiting peritoneal dissemination. They speculated that exosome CD47 may be a favorable therapeutic target for ovarian cancer. CD47 plays an important role in cell functional behavior and immune homeostasis related to cancer prognosis (Fig. 2 in Supplementary material) [19].

## The involvement of CD47 in ovarian cancer immunotherapy

As a macrophage immune checkpoint, CD47 can interact with Sirpα on macrophages to provide a “don’t eat me” signal, bind to and block Sirp-mediated phagocytosis of tumor cells [20, 60, 61]. Sirpα, the most studied ligand of CD47, is an inhibitory receptor expressed on myeloid cells. It is mainly expressed in macrophages, dendritic cells and neurons [21, 62]. The cytoplasmic domain of Sirp contains tyrosine motifs that are phosphorylated and recruit inhibitory molecules. We can see it intuitively in Fig. 2, the combination of Sirp and CD47 can coupling Sirp with phosphatase, thus preventing myosin IIA from aggregating to form phagocytic synapses and inhibiting phagocytic action [63]. The CD47/Sirpα checkpoint was first discovered in 1999 [64]. Inhibition of CD47 signaling has been proved to be to induce Sirpα-dependent phagocytosis, thereby enhancing the phagocytosis activity of innate cells against cancer cells and targeting the innate immune regulatory system [65, 66]. CD47/Sirpα blocking has emerged as the next generation of immune checkpoint interference strategies in various malignancies following programmed death 1 (PD-1)/ programmed cell death-ligand 1 (PD-L1).

Oncolytic viruses (OV) are a new class of drugs that have the ability to selectively replicate in tumor cells and lyse targets through a variety of mechanisms, including cytotoxic cytokine directed oncolytic, tumor vascular targeting, and bystander effects [67, 68]. OV has a recognized application in cancer immunotherapy [69–73]. As an ideal vector, OV can be payload specific delivered into the tumor environment after administration [74]. More importantly, infection with OV can significantly activate the immune response in the local tumor microenvironment, thereby increasing the effectiveness of antibodies or other payloads delivered by OV [67, 75]. Preclinical data indicated that the combination of OV and other therapeutic methods can have a synergistic effect to optimize the therapeutic effect [76–79]. OV therapies and monoclonal antibody (mAb) therapies are emerging as attractive therapeutic agents for the treatment of cancer. Systemic administration of IgG1 anti-CD47 mAb, blocking the “don’t eat me” pathway, was associated with severe toxicity. In order to improve the therapeutic effect while reducing the toxic side effects, Tian and Xu et al. designed an oncolytic herpes virus (oHSV) (Fig. 2). Full-length soluble anti-CD47 mAb was expressed using a human IgG1 scaffold (OV-αCD47-G1) or IgG4 scaffold (OV-αCD47-G4). IgG1 and IgG4 anti-CD47 mAb secreted by oHSV-infected tumor cells blocked the CD47/Sirpα signaling pathway and enhanced the phagocytosis of ovarian tumor cells by macrophages. OV-αCD47, especially OV-αD47-G1, can improve survival in xenograft and immunocompetent mouse models of ovarian cancer by activating NK cytotoxicity and strengthening macrophage phagocytosis. This study explored evidence that oHSV encoding full-length human IgG1 anti-CD47 mAb can exert its known oncolytic function by augmenting innate immunity, when used alone or in combination with other agents. And it can regulate immune cells, thereby improving the treatment of ovarian cancer (Fig. 2 in Supplementary material) [80].

OV carrying therapeutic transgenes have shown great potential in cancer immunotherapy. An oncolytic adenovirus carrying Sirpα-IgG1 Fc fusion gene (SG635-SF) has been constructed. The virus blocks the “don’t eat me” signal of CD47 in cancer cells. In this study, 5/35 chimeric fibers were applied to improve infection efficiency. In SKOV3 xenograft tissues, CD47 was blocked, and macrophage infiltration was significantly increased, which was not observed in CD47-negative HepG2 cells. The strong enhancement of SG635-SF antitumor effect was thus demonstrated to be CD47-dependent, suggesting the efficacy of SG635-SF in the treatment of CD47-positive cancers (Fig. 2 in Supplementary material) [81]. This evidence suggests that this novel oncolytic virus is very promising for cancer immunotherapy.

With the significant progress in nanotechnology, nanoplatforms (NPS) can assist in precision cancer treatment with improved anti-tumor efficacy and reduced side effects [82–84]. At present, liposomes have become one of the most popular delivery vectors. Liposome delivery can improve the solubility of poorly water-soluble antitumor drugs, thereby improving the existing cancer treatment plan [85–88]. However, liposomes are easily cleared by mononuclear phagocyte system (MPS), which limits their wide application in cargo delivery [89, 90]. Exosomes are small nanovesicular vesicles that can also serve as delivery vehicles. Therapeutic drugs such as small molecules or nucleic acid drugs can be incorporated into exosomes, and then delivered to specific types of cells or tissues to achieve targeted drug delivery. Targeted drug delivery can increase local drug concentration and reduce side effects [91–94]. Therefore, exosomes have been favored by more and more researchers. However, most natural exosomes have a short biological half-life in vivo (< 6 h). In addition, there are content components limited to parental cells, which are not loaded with drug molecules and have limited therapeutic effects [95, 96]. Because CD47/Sirpα binding initiates the "don't eat me" signal that inhibits phagocytosis [97–99]. Transfection of exosomes with CD47 could protect exosomes from phagocytosis by macrophages [89]. The study has pointed out that hybrid nanoparticles formed by membrane fusion of engineered exosomes (transfected with CD47 gene) and liposomes can deliver drugs and avoid clearance by MPS system (Fig. 2) [56]. Composite nanovesicular vesicles possess the excellent properties of extracellular vesicles and liposomes, such as good biocompatibility, high loading capacity, excellent targeting, and immune escape of MPS [100, 101]. Li and He et al. found that miR497 has a low transcription efficiency, whereas triptolide (TP) has severe systemic toxicity and weak water solubility. To investigate whether the combined application of miR497 and TP could further overcome ovarian cancer chemotherapy resistance through synergistic inhibition of mTOR signaling pathway, they prepared ph-sensitive bionic targeted hybrid nanoparticles called HENPs. The biological hybrid nanoparticles were synthesized by fusion of CD47-expressing tumor exosomes and tumor-targeting peptide cRGD (cyclic arginine-glycine-aspartic acid) modified liposomes (miR497/ TP-henPs), which were responsible for the delivery of miR497 and TP. Under the acidic conditions of the tumor microenvironment, biotin helical nanoparticles can rapidly cleave and release miR497 and TP, as well as synergistically induce ovarian cancer cell apoptosis by inhibiting the PI3K/AKT/mTOR signaling pathway. TP depleted glutathione in tumor cells and elevated intracellular reactive oxygen species (ROS) in order to promote tumor cell death. Finally, TP overcomes ovarian cancer resistance by regulating macrophage polarization. The study found that the mixed nanoparticles have a wide range of uses with very low toxicity to normal tissues. The significance of this finding rests on the fact that nanoparticle encapsulation maybe can help solve the dilemma of chemotherapeutic drugs (Fig. 2 in Supplementary material) [102]. This would be a promising approach to overcome cisplatin resistance in ovarian cancer.

TSP-1 is a 450 kDa homotrimer glycoprotein that is overexpressed in many solid tumors [103, 104]. It belongs to a family of secretory proteins that regulate cell behavior by binding to molecules in the extracellular matrix and receptors on the cell surface [105]. High affinity binding of the C-terminal domain of TSP-1 to cells requires CD47 [106]. Therefore, CD47 acts as a receptor for TSP1 signal expressed on T cells, and they have a strong affinity (Fig. 2) [66]. As a key signal axis, they play a certain role in the tumors' occurrence and development, as well as the resistance to chemoradiotherapy [21, 107, 108]. The literatures have indicated that the combination of TSP1 and CD47 can severely inhibit T cell differentiation, activation and proliferation, regulate adaptive immunity, and weaken macrophage activation [109, 110]. As a result, strategies aimed at blocking the TSP-1/CD47 axis are currently becoming the focus of innovation in cancer therapy.

TSP-1 is not only an important extracellular matrix glycoprotein, but also a naturally occurring angiogenesis inhibitor with powerful antitumor effects [111]. This protein contains three homologous platelet reactive protein type 1 repeat domains (3TSR) that bind to the CD36 receptor on endothelial cells and have most antiangiogenic effects [112]. 3TSR can directly induce apoptosis of ovarian cancer cells through CD36-dependent mechanism [113, 114]. As a small bioactive recombinant peptide, 3TSR has been shown to have significant antitumor effects in various models either alone or in combination with chemotherapy [112, 115–117]. And it has significant advantages over other antiangiogenic therapies that target only VEGF ligands or receptors. Although these recombinant proteins/peptides are effective in preclinical ovarian cancer models, their relatively short half-life makes them easy to clear from circulation and require daily injection to maintain biological activity [113]. Adeno-associated viruses (AAV) are a class of linear single-stranded uncoated DNA viruses that can be engineered to deliver DNA to target cells [118, 119]. AAV vector has many advantages, such as sustainable expression, wide host range, site-specific integration and no obvious pathogenicity, which can be widely used in scientific research and clinical gene therapy (Fig. 2) [120, 121]. In a preclinical immunoactive ovarian cancer mouse model, AAV gene therapy vectors were used to express 3TSR alone or in combination with the CD47-binding peptide of TSP-1 to assess its effect on tumor development and survival. This method can continuously produce antiangiogenic proteins from within or near the tumor, which may be more effective in controlling tumor growth [114, 122]. To evaluate the effect of AAV-mediated angiogenic protein expression in vivo, 1 × 10^11^vg of each AAV vector was intraperitoneally injected into ID8 ovarian tumor mice 60 days after ID8 cell implantation. Treatment of ID8 tumor-bearing mice with AAV vectors expressing 3TSR, CD47 binding peptide, or 3TSR + CD47 binding peptide resulted in a significant reduction in the size of the primary tumor and, in some cases, in the number of secondary lesions. On day 60, C57BL/6 ID8-bearing mice were intraperitoneally injected with 500 μl of 1 × 10^11^vg AAV vector to express secreted 3TSR, CD47-binding peptide, 3TSR + CD47-binding peptide, and GFP. The goal was to evaluate the effect of AAV-mediated expression of angiogenic proteins in vivo. The study ended with the determination of signs of ascites accumulation and poor physical condition in mice. It was also noted that only AAV-mediated 3TSR expression significantly increased the median survival rate in the ovarian cancer mouse model. This study demonstrated that AAV-mediated expression of 3TSR is safe and effective in inhibiting tumor progression, and may provide a new, minimally invasive approach for the treatment of ovarian cancer (Fig. 2 in Supplementary material) [123].

Plant virus nanoparticles (VNPs) have been explored as a unique class of nanocellulators for biomedical applications. It has the advantages of low production cost, safety and degradability, and can be used as an effective treatment [124–126]. The Cowpea Mosaic Virus (CPMV) belongs to the genus Comovirus. CPMV is an icosahedral virus approximately 27 nm in diameter [127]. It has been reported that a variety of its molecules are able to attach well to envelope proteins. The five reactive lysine residues of the CPMV coat protein provide sites for chemical binding to a variety of compounds, such as fluorescent dyes [128]. CPMV is one of the most developed VNPS in biomedical and nanotechnology applications, with the ability to target specific tissues and act as an effective drug delivery system (Fig. 2) [129, 130]. ID8-Defb29/Vegf-A is a highly aggressive ovarian tumor cell line that overexpresses mouse vascular endothelial growth factor-A (Vegf-A164) and β-defensin-29 (Defb29). Immature dendritic cells can mediate tumor angiogenesis through the synergistic effect of the two. This is the main reason why intraperitoneal ID8-Defb29/Vegf-A tumors lead to rapid ascites accumulation and shortened survival [131, 132]. Wang et al. used CPMV orthotopic inoculation and CD47 blocking antibody to inoculate ID8-Defb29/Vegf-A cells intraperitoneally (i.p.) in C57BL/6 mice and performed combined immunotherapy experiments. They observed that CD47 was highly expressed in this tumor cell line. Moreover, single treatment could promote the phagocytosis of macrophages to tumor cells. However, the combination of CD47 Ab and CPMV has a synergistic potential to induce tumor cell death by activating macrophages compared to monotherapy. Although CPMV showed anti-tumor effects, CD47 Ab appeared to be ineffective in the treatment of C57BL/6 mice by intraperitoneal injection. High doses of CD47Ab delayed intraperitoneal tumor development but did not increase survival. In addition, mice treated with higher doses of CD47 Ab developed more severe malignant ascites and weight gain (Fig. 2 in Supplementary material) [66]. Therefore, in the ID8-Defb29/Vegf-A model, blocking the CD47 axis to turn off the “don’t eat me signal” is not sufficient to slow tumor progression and achieve durable anti-tumor effects, especially durable adaptive immune responses. This work suggested a novel strategy to promote macrophage activity to kill tumor cells. In the future, it is expected to enhance T cell-targeted immunotherapy by inducing the innate and adaptive arms of the immune system.

Immunotherapy using engineered T cells modified with chimeric antigen receptors (CAR) has proven to be a promising treatment for hematologic cancers and solid tumors [133–136]. Macrophages play an important role in recognizing and consuming damaged and aged cells [137]. Thus, targeting multiple antigens may improve the effectiveness of CAR immunotherapy (Fig. 2). The previous study has used a single-chain variable fragment (ScFv) derived from a mouse CD47 antibody to generate CD47-CAR-T cells targeting different cancer cell lines. Humanized mouse CD47 ScFv could bind to CD47 antigen efficiently. Moreover, humanized CD47-CAR-T cells specifically killed ovarian, pancreatic, and cervical cancer cell lines, and produced CD47-related IL-2. This report showed high specificity of CD47-CAR-T cells against CD47-positive cancer cells and no CAR-T activity in target cells with low CD47 expression (Fig. 2 in Supplementary material) [138]. Thus, there exists a therapeutic window in which CD47-CAR-T cells affect only highly expressed CD47 cells. This is the first report of a novel approach to target cancer cells with CD47-CAR-T cells. It demonstrated the high potency of CD47-CAR-T cells against cancer cells in vitro and provided a novel anticancer cell therapy. CD47 is ubiquitously overexpressed in multiple tumor types. But it is also expressed on many normal cells [139]**.** Shu et al. generated dual CAR-T cells targeting two tumor antigens: tumor-associated glycoprotein 72 (TAG-72) and CD47. Investigators designed a truncated CD47 CAR without an intracellular signaling domain and monomeric it to reduce damage to normal tissues. CD47 CAR helps to bind to CD47^+ ^cells and has the prospect of being able to eliminate TAG-72^+^ cells by TAG-72 CAR. The results suggested that co-expression of TAG-72 CAR and CD47-truncated monomeric CAR on T cells may be an effective dual CAR-T cell strategy for the treatment of ovarian cancer. This strategy can also be applied to other adenocarcinomas (Fig. 2 in Supplementary material) [140].

To date, many efforts have been made to block CD47/Sirpα to promote cancer cell phagocytosis [98, 141, 142]. However, due to the universal CD47 expression, antibody blockade interferes with the important physiological role of its receptor and prompts phagocytes to induce adverse events, such as severe anemia, thrombocytopenia, and splenomegaly [143]. For the above reasons, Jeanne and Sarazin et al. prepared the first ever orthotopic antagonist TAX2 that is targeted to TSP-1 and selective for TSP-1/CD47 interaction [23]. It is able to bind the C-terminal domain of TSP-1 to selectively disrupt the binding of TSP-1 to CD47 (Fig. 2) [144]. Some studies have pointed out that TAX2 has anti-cancer properties [145, 146]. By constructing subcutaneous nude mice model and advanced ovarian cancer metastasis model, it was found that tumor growth was significantly inhibited in TAX2 treated animals. Moreover, TAX2 treatment could increase the number of infiltrating CD4^+^T lymphocytes and stimulate deeper infiltration of T cells within the tumor, leading to the inhibition of tumor growth. In the study of combined treatment of TAX2 peptide and anti-PD-1 mAb, it was observed that the tumor growth inhibition effect of TAX2 and anti-PD-1 treatment was similar, but the combined treatment of the two showed a better inhibitory effect. Moreover, the combination of TAX2 peptide and anti-PD-1 mAb could significantly inhibit ascites production and metastatic dissemination. Further experimental results showed that the TAX2 peptide did not reproduce the adverse events associated with anti-CD47 therapy, and neither TAX2 monotherapy nor in combination with anti-PD-1 mAb had any effect on platelet activation capacity. The authors suggested that the immune mode of action of TAX2 peptide is most dependent on adaptive immunity, and this mode does not affect the binding of CD47 to its macrophage counterreceptor Sirpα. This study is the first to characterize the antiangiogenic properties of a TAX2 peptide using in vitro, ex vivo, and in vivo models. The relevance of targeting the TSP-1: CD47 axis in ovarian cancer was confirmed, enabling simultaneous targeting of different components of the ovarian microenvironment with a single molecule (Fig. 2 in Supplementary material) [23]. In the future, it is expected to be a new cancer immunotherapy agent and further explored in ovarian cancer immunotherapy.

## Clinical studies targeting CD47

Great progress has been made in targeting CD47 for cancer immunotherapy in solid tumors and hematological malignancies. By July 27, 2023, there were 95 clinical trials related to CD47, of which 35 were available for ovarian cancer patients. From Table 1 we can notice that a number of therapeutic products targeting CD47 are in development, including anti-CD47 mAb, bispecific antibodies (BsAbs) that target CD47 and other molecules, as well as Sirpα-related fusion proteins. Several therapeutic products have shown promising results in clinical studies.

**Table 1:** Clinical studies of CD47 associated with ovarian cancer

NCT number	Status	Conditions	Primary interventions	Isotype	Mechanism of action	Phase	Enrollment	Study start	Primary completion	Study completion
NCT04349969	Completed	Neoplasms malignant	AK117	IgG4	Humanized anti-CD47 mAb	1	50	2020/4/23	2022/11/8	2022/11/8
NCT02216409	Completed	Solid tumor	Hu5F9-G4	IgG4	Humanized anti-CD47 Ab	1	88	2014/8	2018/10	20018/12
NCT03558139	Completed	Ovarian cancer	Hu5F9-G4	IgG4	Humanized anti-CD47 Ab	1	34	2018/5/23	2020/12/3	2020/12/3
NCT04097769	Completed	Advanced solid tumor	HX009	IgG4	Humanized anti-CD47/PD-1 BsAb	1	21	2019/6/12	2021/3/18	2022/9/29
NCT03717103	Completed	Advanced malignancies	IBI188	IgG4	Humanized anti-CD47 mAb	1	49	2019/1/10	2022/2/16	2022/2/16
NCT03763149	Completed	Advanced malignancies	IBI188	IgG4	Humanized anti-CD47 mAb	1	20	2019/2/19	2021/3/29	2021/3/29
NCT03512340	Completed	Advanced solid cancers, hematologic cancers	SRF231	IgG4	Humanized anti-CD47 mAb	1	148	2018/3/13	2020/9/15	2020/9/29
NCT05467670	Recruiting	Ovarian cancer	ALX148	IgG4	Humanized anti-CD47 mAb	2	31	2022/12/16	2025/9	2027/12
NCT05868226	Recruiting	Solid tumor and17 more	ALX148	IgG4	Humanized anti-CD47 mAb	1	40	2022/12/22	2026/12/30	2027/12/30
NCT05767060	Recruiting	Advanced malignant tumor	BAT7104	–	Recombinant anti-PD-L1/CD47 BsAb	1	42	2022/1/20	2024/1/20	2024/1/20
NCT05200013	Recruiting	Advanced solid tumors	BAT7104	–	Recombinant anti-PD-L1/CD47 BsAb	1	29	2022/4/29	2023/4/30	2024/12/31
NCT05765851	Recruiting	Advanced solid tumor, breast cancer	DS-1103a	IgG4	Recombinant humanized anti-Sirpα Ab	1	78	2023/5/30	2026/6/30	2026/6/30
NCT05221385	Recruiting	Solid tumor, non-hodgkin lymphoma	Gentulizumab	–	Anti-CD47 mAb	1	58	2021/4/12	2024/5/30	2024/5/30
NCT05429008	Recruiting	Advanced tumors	HMPL-A83	–	Humanized anti-CD47 mAb	1	99	2022/7/15	2024/2	2025/9
NCT05276310	Recruiting	Advanced cancer	IMC-002	IgG4	Humanized anti-CD47 mAb	1	24	2022/5/10	2024/7/31	2024/12/31
NCT05780307	Recruiting	Advanced solid tumor, and 5 more	IMM2520	–	Recombinant CD47/PD-L1 BsAb	1	48	2023/3/23	2024/3/10	2025/10/26
NCT05076591	Recruiting	Advanced solid tumor and 2 more	IMM2902	–	recombinant CD47/HER-2 BsAb	1	135	2022/6/20	2023/12	2024/12
NCT05615974	Recruiting	Malignant tumors	LM101	–	Sirpα mAb	1, 2	145	2023/1/11	2024/12	2025/8
NCT05261490	Recruiting	Ovarian cancer, ovarian neoplasms, ovarian carcinoma, 3 more	PF-07901801	IgG4	Sirpα-Fc Fusion Protein	1, 2	50	2022/8/1	2024/12/4	2025/12/4
NCT05403554	Recruiting	Epithelial ovarian cancer, and 2 more	NI-1801	–	Humanized anti-CD47/mesothelin BsAb	1	40	2022/4/29	2025/6/30	2025/9/30
NCT04900519	Recruiting	Solid tumor, relapsed solid neoplasm, refractory tumor	STI-6643	IgG4	Humanized anti-CD47 mAb	1	100	2021/11/24	2024/12	2025/3
NCT05192512	Recruiting	Advanced cancer	TQB2928	-	Anti-CD47 mAb	1	180	2022/1/24	2024/1	2025/1
NCT03834948	Active, not recruiting	Solid tumor	AO-176	IgG2	Humanized anti-CD47 mAb	1, 2	183	2019/2/4	2023/5	2023/5
NCT05731752	Active, not recruiting	Advanced solid tumors	HX009	IgG4	Humanized anti-CD47/PD-1 BsAb	1	25	2020/6/4	2023/11/4	2024/10/28
NCT04886271	Active, not recruiting	Advanced solid tumor	HX009	IgG4	Humanized anti-CD47/PD-1 BsAb	2	210	2021/5/12	2022/12/10	2023/2/10
NCT04912466	Active, not recruiting	Advanced solid tumor	IBI322	IgG4	Humanized anti-CD47/PD-L1 BsAb	1	36	2021/6/20	2023/3/20	2023/9/20
NCT04328831	Active, not recruiting	Advanced malignancies	IBI322	IgG4	Humanized anti-CD47/PD-L1 BsAb	1	218	2020/7/31	2023/9	2023/12
NCT04881045	Active, not recruiting	Ovarian cancer and 2 more	PF-07257876	–	CD47-PDL-1 antibody	1	28	2021/8/18	2023/10/27	2023/10/27
NCT05957536	Not yet recruiting	Her-2 positive advanced solid tumors	D3L-001	–	CD47/HER-2 BsAb	1	110	2023/8	2026/3/11	2026/3/11
NCT05892718	Not yet recruiting	Advanced solid tumor, refractory non-hodgkin lymphoma	HCB101	–	Sirpα-Fc fusion protein	1	60	2023/8/18	2025/5/30	2025–11-15
NCT03957096	Terminated	Ovarian carcinoma and 7 more	SGN-CD47M	–	Humanized CD47/MER6 mAb	1	16	2019/7/17	2020/9/14	2020/9/14
NCT02890368	Terminated	Solid tumors, and 6 more	TTI-621	IgG1	Sirpα-Fc Fusion Protein	1	56	2016/9	2020/3/31	2020/3/31
NCT04306224	Unknown status	Solid tumor, lymphoma	IMC-002	IgG4	Humanized anti-CD47 mAb	1	24	2020/6/5	2022/5	2022/12
NCT04588324	Unknown status	Solid tumor	SHR2150	IgG4	ISelective TLR7 Agonist	1, 2	50	2020/10/10	2021/11/30	2022/11/30
NCT04854681	Unknown status	Advanced solid tumors and hematological malignancies	TQB2928	–	Anti-CD47 mAb	1	20	2021/8/1	2022/7/1	2022/12/1

Hu5F9-G4 (5F9) is a humanized IgG4 mAb with high antitumor activity in a wide range of solid-tumor hematologic malignancies [147–149]. Studies have reported first-in-human phase I trials of 5F9 in patients with advanced solid tumors and lymphomas, including 13 patients with ovarian cancer. In this study, the most common toxicities that patients experienced were targeted, mild, transient, and predictable anemia. Most 5F9 related adverse events (AE) were mild to moderate in severity. At dose levels of 20 mg/kg or higher, common adverse events during infusion were headache, fatigue, fever, and chills. Despite widespread CD47 expression on normal tissues, 5F9 was well tolerated by the patient. This may be because normal cells, unlike malignant cells, lack phagocytic signals and are less susceptible to CD47 blockade. The objective response rate was 5.2 months in a patient with clear cell ovary and 9.2 months in a patient with fallopian tube cancer. Patients had reductions in target lesions of 50% and 44%, respectively. They all had a significant reduction in the CA125 tumor marker. This study demonstrated that inhibition of the CD47/Sirpα innate immune checkpoint is safe. The targeting capability of CD47 could be an immunotherapy strategy for the treatment of human cancers [150]. Another clinical trial of Magrolimab (Hu5F9-G4) combined with anti-PD-L1 avelumab, which began in 2018, involved 34 patients. Patients with ovarian, fallopian tube and primary peritoneal cancer that had progressed within 1 to 6 months after prior platinum-based chemotherapy were eligible for treatment. The safety and tolerability of Magrolimab combined with avelumab in the treatment of advanced solid tumors and its antitumor activity were further evaluated, but the results have not yet been published (NCT03558139).

For patients with platinum-resistant ovarian cancer who are ineligible for chemotherapy plus bevacizumab, pegylated liposomal doxorubicin (PLD) is the care’s standard. However, the clinical benefit in this patient population is modest. Two separate phase II trials for patients with this type of ovarian cancer were initiated in August 2022. A clinical study with TTI-622 combined with PLD 40 mg/m^2^ in platinum-resistant ovarian cancer, including ovarian, peritoneal, and fallopian tube malignancies, is being recruited. And a dose-expansion cohort for the combination regimen is established for further evaluation. The goal of this clinical trial was to improve the activity of PLD in a safe manner to provide a more effective treatment option for this patients’ group. This is a multicenter, open-label study evaluating the combination of TTI-622 and pegylated liposomal doxorubicin in patients with platinum-resistant ovarian cancer (NCT05261490). In an unrecruited trial, the immune checkpoint inhibitor pembrolizumab, CD47 inhibitor ALX148, and liposomal doxorubicin were evaluated for safety and efficacy in patients with recurrent platinum-resistant ovarian cancer (NCT05467670). The results of these two clinical trials are promising and will lead to an effective treatment option for patients with clinically platinum-resistant ovarian cancer.

The targeting of CD47 is an immunotherapy strategy for the treatment of human cancer. It has become another highly competitive target in cancer immunotherapy after PD-1/PD-L1, and has been used in a variety of clinical trials. There will hopefully be several CD47-related drug candidates that will emerge and be applied to the clinic to meet the urgent needs of patients.

## Conclusion and future perspectives

In recent years, the addition of PARP inhibitors and anti-angiogenic bevacizumab has led to improved treatment prospects and survival outcomes for ovarian cancer patients [151]. However, the risk of disease recurrence remains high after first-line treatment [152]. Therefore, there is a need to find effective and safe treatments. Immunotherapy is a widely studied and innovative strategy. It can effectively control and eliminate tumors by restarting and maintaining the tumor immune cycle, thereby restoring normal antitumor immune responses [153].

Immune checkpoint inhibitors are the most promising treatment for incurable tumors [154], including ovarian cancer [155–157]. Today, the best known and widely used checkpoints include T cell surface molecules such as cytotoxic T lymphocyte antigen 4 (CTLA-4) and PD-1 [158, 159]. However, immune checkpoint inhibitors often cause dose-dependent side effects and immunotoxicity. In addition, their response rates and effective rate in ovarian cancer are low [52]. As a promising immunotherapy, the most common side effect of CD47 immune checkpoint blockade agents is hematotoxicity [160]. In order to overcome the adverse events caused by targeting CD47, researchers have used delivery vector technology, transgenic technology, nanotechnology, and combination therapy in pre-clinical trials in order to activate the immune response, improve the treatment effect, and reduce the incidence of side effects. Each study has obtained varying degrees of promising results. In the phase I clinical trial of Sikic and Lakhani et al., the severity of most 5F9-related AE was mild to moderate, and no dose-related thrombocytopenia or malnutrition was observed in the experimental results. This experiment showed that 5F9 can be directly infused in the outpatient clinic, which can improve the convenience of patients to visit a doctor. Moreover, pretreatment and slow instillation can reduce the occurrence of other adverse reactions [150]. However, the number of patients with ovarian cancer was small because of the heterogeneity among tumors. Therefore, more clinical studies are needed to confirm the efficacy and safety of this immune checkpoint blockade agents in the treatment of ovarian cancer patients. Most immune-related side effects are manageable. It is hoped that detailed clinical monitoring of patients by researchers, early assessment and pretreatment of potential side effects, as well as timely treatment of early symptoms, will improve the authenticity and reliability of clinical trials [52]. In the future research, we hope to find complementary advantages of targeting CD47 combined with different immunotherapy/non-immune mechanisms. Through the use of multi-omics analysis, single-cell technology, 3D organoid model and other pre-clinical models of ovarian cancer, as well as more advanced experimental methods, CD47 related mechanisms are further studied and verified. Targeting CD47-associated immune checkpoints will be applied to clinical practice. Moreover, with the upstream and downstream of CD47 related signaling pathway being completely blocked, more and more excellent clinical treatment programs will be produced and play an important role in the treatment of ovarian cancer in the future.

### Supplementary Information

Below is the link to the electronic supplementary material.

Supplementary file1 (DOCX 1054 KB)

## Data Availability

Not applicable.

## Abbreviations

3TSR: Three homologous platelet reactive protein type 1 repeat domains
5F9: Hu5F9-G4
AAV: Adeno-associated viruses
AE: Adverse events
ALDH: Acetaldehyde dehydrogenase
ALDHigh: High ALDH1 expression
anti-CD47 mAb: Anti-CD47 monoclonal antibody
BsAbs: Bispecific antibodies
CAR: Chimeric antigen receptors
CD47: Antibody (CD47 Ab)
CSCs: Cancer stem cells
CTLA-4: Cytotoxic T lymphocyte antigen 4
Defb29: β-Defensin-29
EAOC: Endometriosis-associated ovarian cancer
EMT: Epithelial-mesenchymal transition
IAP: Integrin-associated protein
mAb: Monoclonal antibody
MPS: Mononuclear phagocyte system
NPS: Nanoplatforms
OCCC: Clear cell carcinoma
oHSV: Oncolytic herpes virus
OS: Overall survival
OV: Oncolytic viruses
OV: Oncolytic viruses
OV-αCD47-G1: IgG1 scaffold
OV-αCD47-G4: IgG4 scaffold
PARP: Poly ADP-ribose polymerase
PD-1: Programmed death 1
PD-L1: Programmed cell death-ligand 1
PFS: Progression free survival
PLD: Pegylated liposomal doxorubicin
ROS: Reactive oxygen species
SCA: Stem cell antigen
ScFv: Single-chain variable fragment
SG635-SF: SIRPα-IgG1 Fc fusion gene
Sirpα: Signal regulatory protein alpha
TAG-72: Tumor-associated glycoprotein 72
TIC: Tumor infiltrating immune cell
TISCH: Tumor Immune Single-cell Hub
TME: Tumor microenvironment
TP: Triptolide
TSP-1: Thrombosponin-1
Vegf-A164: Vascular endothelial growth factor-A
VNPs: Virus nanoparticles
