# Supplementary material for: The biological roles of CD47 in ovarian cancer progression
Source: Cancer Immunol Immunother. 2024 Jun 4;73(8):145. doi: 10.1007/s00262-024-03708-3 (PMC11150368; doi:10.1007/s00262-024-03708-3)
Supplement: Supplementary file 1 — Supplementary file1 (DOCX 1054 KB) [file 262_2024_3708_MOESM1_ESM.docx]

**
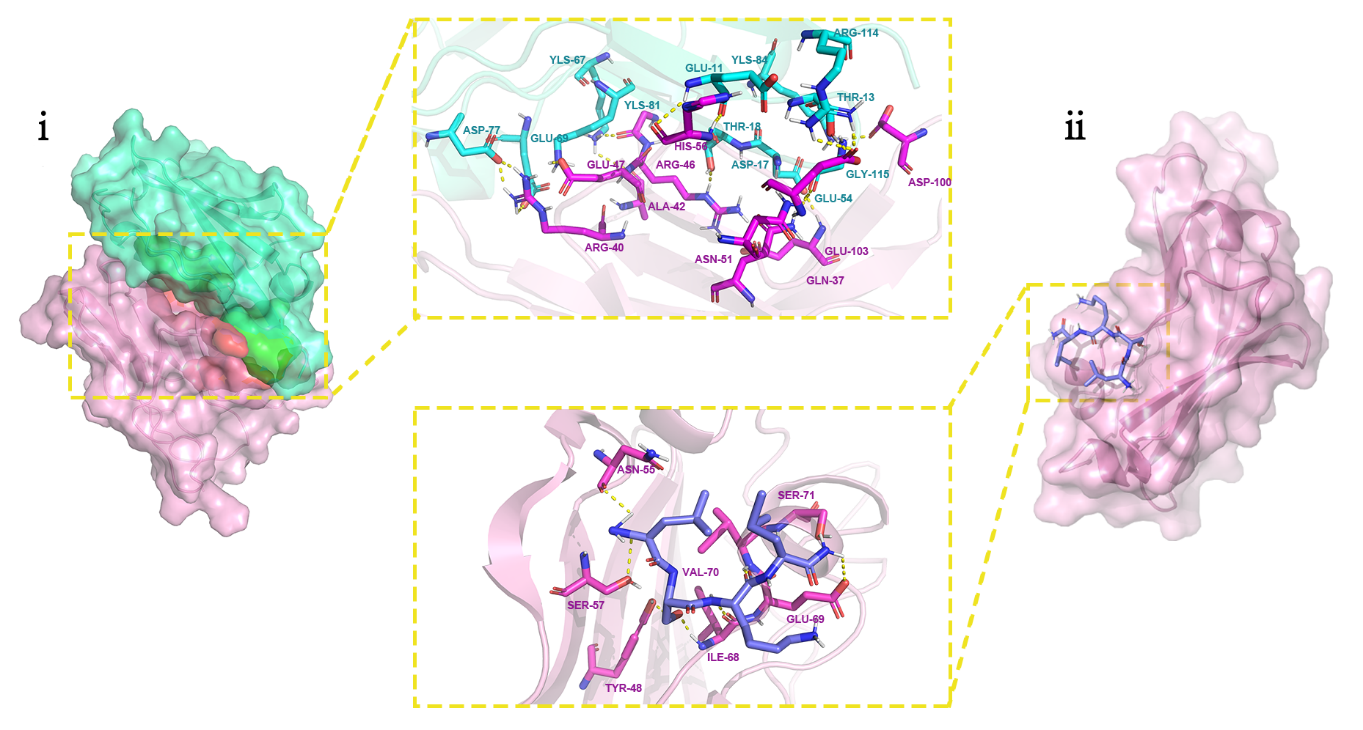
**

**Fig. 1 Structure of CD47 and its interactions with SIRP-α and TSP-1.** PyMOL was used to visualize the docking process and interaction of CD47 with SIRP-α and TSP-1. The molecular docking procedure is composed of four stages: searching of the binding modes of the receptor and the ligand, evaluating the docked modes with the refined scori, filtering of docked models to eliminate the irrational docked structures,optimizing the structures. i, lgV like domain of CD47 (marked by pink) interacts with SIRP-α (marked by green). ii, The lgV like domain of CD47 (marked by pink) interacts with TSP-1 (marked by purpul).

**
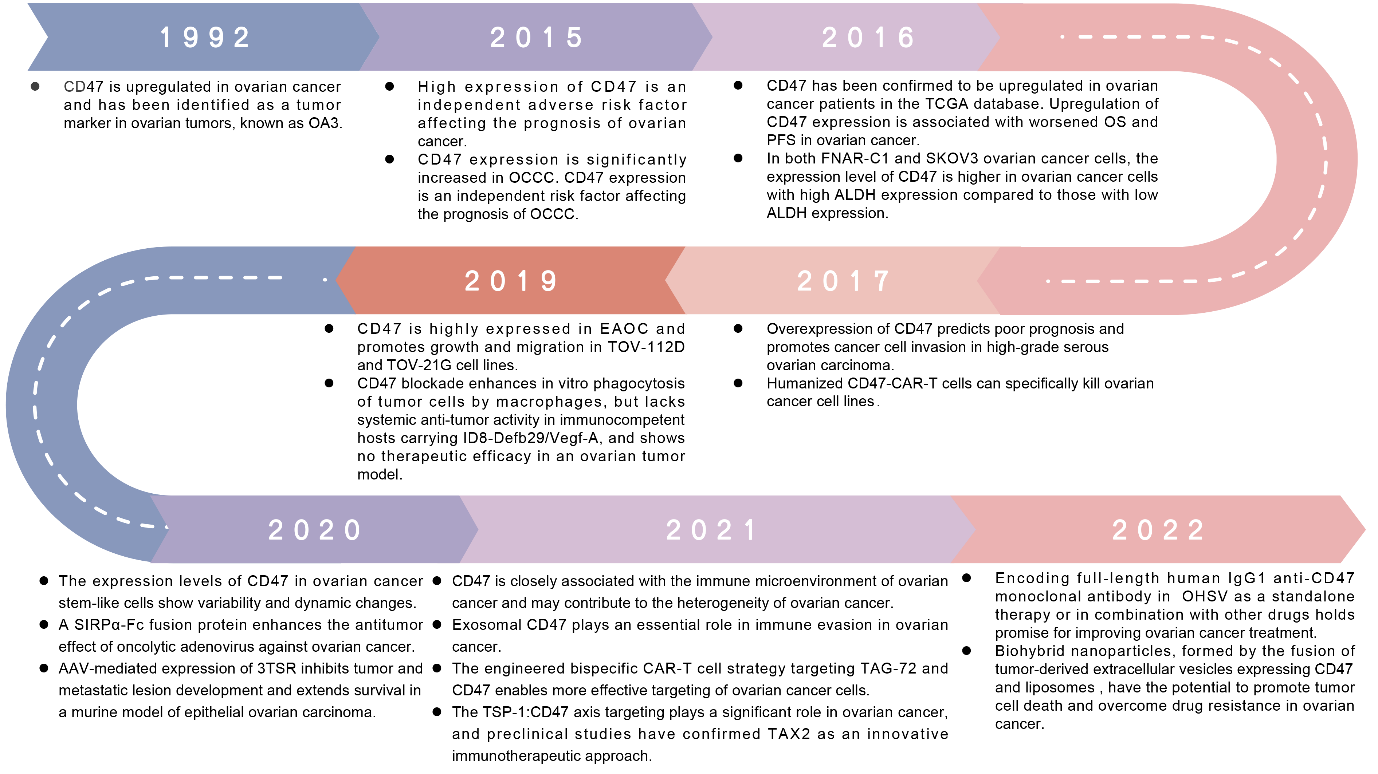
**

**Fig. 2 The preclinical research progress and key findings regarding CD47 in ovarian cancer**
